# Supplementary material for: Mechanical stress through growth on stiffer substrates impacts animal health and longevity in C. elegans
Source: bioRxiv. 2024 Apr 12:2024.04.11.589121. Preprint. [Version 2] doi: 10.1101/2024.04.11.589121 (PMC11030433; doi:10.1101/2024.04.11.589121)
Supplement: Supplement 3 [file NIHPP2024.04.11.589121v2-supplement-3.pdf]

bioRxiv preprint doi: https://doi.org/10.1101/2024.04.11.589121; this version posted April 12, 2024. The copyright holder for this preprint (which was not certified by peer review) is the author/funder, who has granted bioRxiv a license to display the preprint in perpetuity. It is made available under aCC-BY 4.0 International license.

Supplemental Figures and Figure Legends

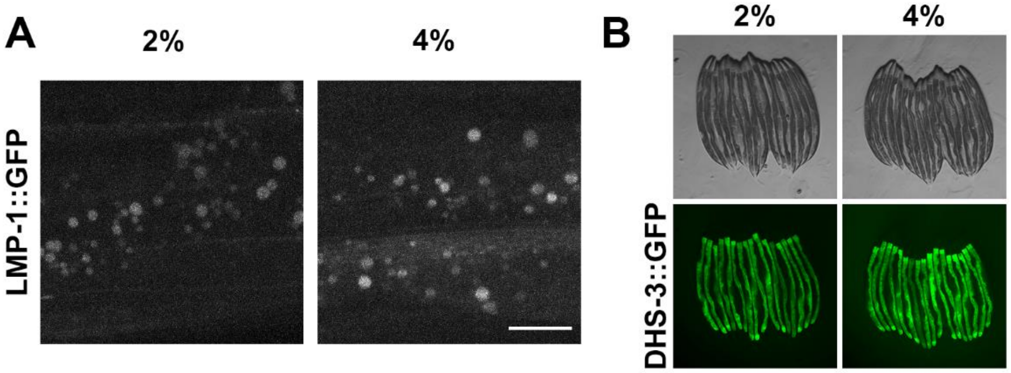

Fig. S1. Growth on stiff substrates has no impact on lysosomes or lipid droplets. (A) Representative max projection fluorescent images of lysosomes by visualization of *lmp-1::GFP*. Images were captured on a Leica Stellaris system using optimized z-slices. **(B)** Representative fluorescent images of lipid droplets by visualization of DHS-3::GFP. For A-B, animals were grown on empty vector (EV) RNAi bacteria from L1 and imaged at day 1 of adulthood. Scale bar is 10 µm. Data is representative of 3 independent trials.

bioRxiv preprint doi: https://doi.org/10.1101/2024.04.11.589121; this version posted April 12, 2024. The copyright holder for this preprint (which was not certified by peer review) is the author/funder, who has granted bioRxiv a license to display the preprint in perpetuity. It is made available under aCC-BY 4.0 International license.

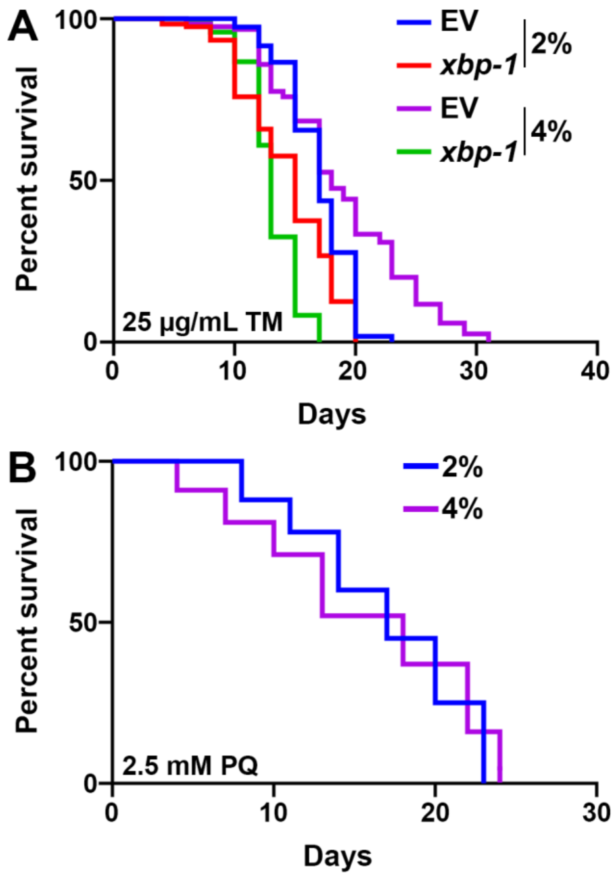

Fig. S2. Growth on stiff substrates increases ER stress resilience. (A) N2 wild-type animals grown on empty vector (EV) or *xbp-1* RNAi on either control (2%) or stiff (4%) agar plates from L1. Animals were transferred onto the same RNAi and agar concentration plates containing 25 μg/mL tunicamycin (TM). Lifespans were scored every 2 days. Data is representative of 3 replicates and statistical analysis is available in **Table S1**. **(B)** N2 wild-type animals grown on EV RNAi on either control (2%) or stiff (4%) agar plates from L1. Animals were moved to plates containing 2.5 mM paraquat (PQ) at day 1 of adulthood and survival was scored every 2 days. Data is representative of 3 replicates and statistical analysis is available in **Table S1**.

bioRxiv preprint doi: https://doi.org/10.1101/2024.04.11.589121; this version posted April 12, 2024. The copyright holder for this preprint (which was not certified by peer review) is the author/funder, who has granted bioRxiv a license to display the preprint in perpetuity. It is made available under aCC-BY 4.0 International license.

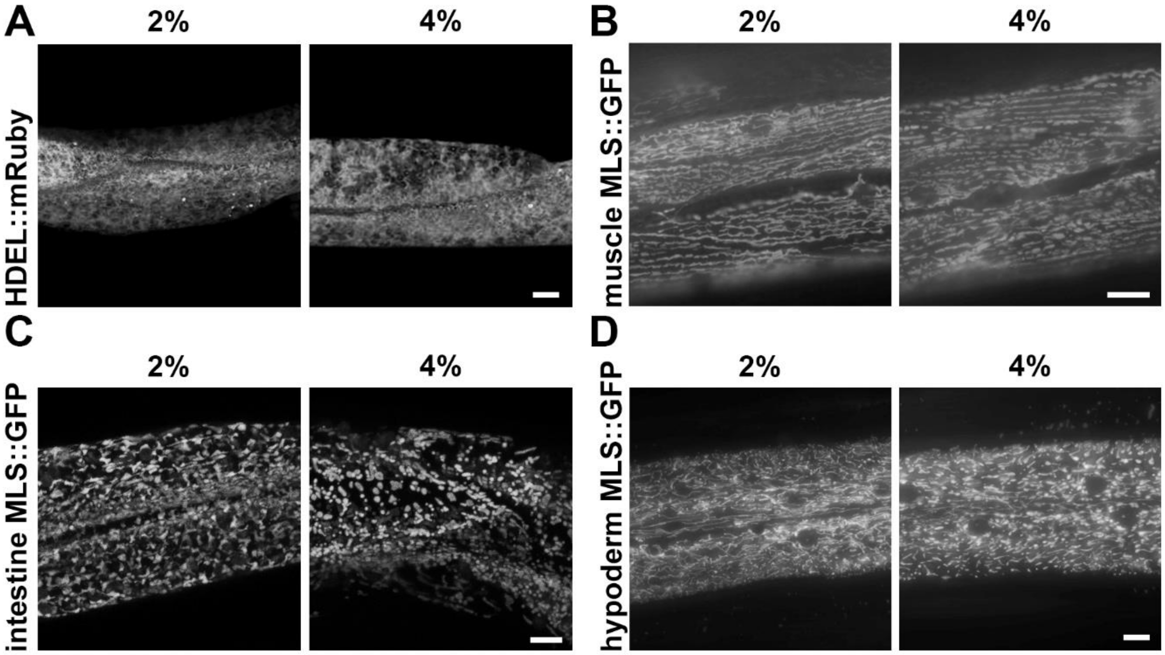

Fig. S3. Growth on stiff substrates does not impact ER or mitochondrial structure. (A) Representative max projection fluorescent images of the endoplasmic reticulum in the intestine by visualization of *vha-6p::HDEL::mRuby*. Images were captured on a Leica Stellaris system using optimized z-slices. Animals were grown on empty vector (EV) bacteria from L1. Animals were moved onto 25 µg/mL tunicamycin containing plates at L4 and imaged at day 1 of adulthood. **(B)** Representative fluorescent images of body wall muscle mitochondria (*myo-3p::MLS::GFP*) are shown. Animals were grown on empty vector (EV) RNAi from L1. Single-slice images were captured on a Leica THUNDER Imager. Scale bar is 10 µm. **(C)** Representative max-projection fluorescent images of intestinal mitochondria (*gly-19p::MLS::GFP*) are shown. Animals were grown on empty vector (EV) RNAi from L1. Z-stack images were captured on a Leica THUNDER Imager using system-optimized z-slices. Scale bar is 10 µm. **(D)** Representative max-projection fluorescent images of hypodermal mitochondria (*col-19p::MLS::GFP*) are shown. Animals were grown on empty vector (EV) RNAi from L1. Z-stack images were captured on a Leica Stellaris using system-optimized z-slices. Scale bar is 10 µm.

bioRxiv preprint doi: https://doi.org/10.1101/2024.04.11.589121; this version posted April 12, 2024. The copyright holder for this preprint (which was not certified by peer review) is the author/funder, who has granted bioRxiv a license to display the preprint in perpetuity. It is made available under aCC-BY 4.0 International license.

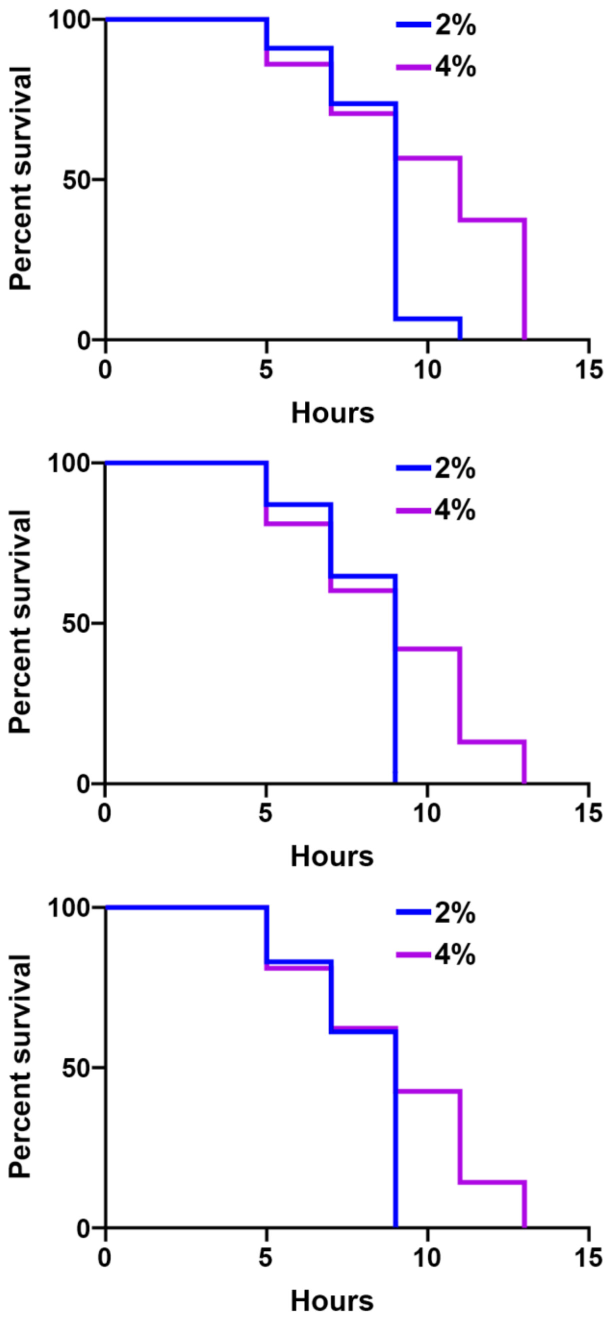

Fig. S4. Growth on stiff substrates results in a mild increase in thermotolerance. N2 wild-type animals grown on EV RNAi on either control (2%) or stiff (4%) agar plates from L1. Animals were moved to 34°C at day 1 of adulthood and survival was scored every 2 hours. All three biological replicates are shown to highlight differences in maximal thermotolerance, despite similarities in median thermotolerance in some replicates. All statistics are available in **Table S1**.
